# Development of Comprehensible Prescription Label Instructions: A Study Protocol for a Mixed-Methods Approach

**DOI:** 10.3389/fphar.2020.00981

**Published:** 2020-07-15

**Authors:** Ekram Maghroudi, Charlotte M. J. van Hooijdonk, Liset van Dijk, Gudule Boland, Channah de Haas, Marleen Journée-Gilissen, Janneke van der Velden, Marcia Vervloet, Henk Westerhof, Jany J. D. J. M. Rademakers, Sander D. Borgsteede

**Affiliations:** ^1^ Department of Patient Information, Health Base Foundation, Houten, Netherlands; ^2^ Department of Family Medicine, Research School CAPHRI, Maastricht University, Maastricht, Netherlands; ^3^ Department of Languages, Literature & Communication, Faculty of Humanities, Universiteit Utrecht, Utrecht, Netherlands; ^4^ Department of Pharmaceutical Care, Nivel, Netherlands Institute for Health Services Research, Utrecht, Netherlands; ^5^ Department of PharmacoTherapy, Epidemiology & Economics (PTEE), Faculty of Mathematics and Natural Sciences, Groningen Research Institute of Pharmacy, University of Groningen, Groningen, Netherlands; ^6^ Department of Prevention and Care for the Chronically Ill Programme, Pharos, Dutch Centre of Expertise on Health Disparities, Utrecht, Netherlands; ^7^ Medicines Information Centre, Royal Dutch Pharmacists Association (KNMP), The Hague, Netherlands; ^8^ Department of Computerization of General Practitioner Care, NHG, The Dutch College of General Practitioners, Utrecht, Netherlands; ^9^ Department of Clinical Decision Support, Health Base Foundation, Houten, Netherlands

**Keywords:** prescription label instructions, comprehensibility, health literacy, mixed-methods, systematic review, textual content analysis, patient studies, implementation

## Abstract

**Introduction:**

Patients receive information about their medication from different sources, including prescription labels. These labels are physically attached to each package dispensed to patients and contain the most important instructions on how to use the medication correctly. However, many patients experience difficulties in understanding and applying the instructions on these labels correctly, especially patients with limited health literacy. The aim of this study is to investigate the comprehensibility of prescription label instructions among patients with adequate and limited health literacy skills, and to implement improvements in primary health care.

**Methods:**

We used a mixed-methods approach, which consisted of four phases. Phase 1 (desk research) was divided into a systematic literature review on the comprehensibility of prescription label instructions (1a) and a content analysis of the textual elements in Dutch prescription label instructions (1b). In phase 2 (patient studies), semi-structured interviews were conducted to investigate the comprehensibility of seven prescription labels among patients with different health literacy skills (2a), and a quantitative study in which the comprehensibility of six optimized prescription labels was compared among patients with different health literacy skills (2b). Patient studies were conducted in eight Dutch pharmacies. In phase 3 optimized prescription label instructions were implemented in national medication databases which has been supported by a guideline (3a), and education of pharmacy workers (3b). Phase 4 consists of evaluating the optimized prescription label instructions by experiences from patients and pharmacists.

**Anticipated Results:**

This mixed-methods approach will result in scientific publications of the individual studies, and a guideline on how to compose comprehensible prescription label instructions to be put on medication packages. Optimized prescription label instructions will be implemented in national medication databases.

**Discussion:**

This protocol describes a mixed-method research to compose and implement comprehensible prescription label instructions and will lead to knowledge about the comprehensibility of textual elements in these labels, with specific attention for patients with limited health literacy. Implementation of optimized prescription label instructions will lead to a better understanding of them, which may contribute to improved medication adherence. A limitation is that non-textual aspects of prescription labels are not investigated.

## Introduction

Instructions on how to use medication are an essential part of patients’ medication management. Patients receive instructions about their medication use from different sources. Dosing instructions are provided by the prescriber, both verbally and written on a prescription. Written information is available in the package insert of the manufacturer and on the package itself (Raynor and Bryant, 2013; van Beusekom et al., 2016). Internationally, the package leaflet is the most common source of written information, with many studies covering this topic, and regulatory requirements concerning the content and comprehensibility of the text (Raynor and Bryant, 2013; Pires et al., 2016; Yuan et al., 2019). These requirements have been developed to ensure the information about medication on the outer package and the package leaflet is accessible, and written in plain language that can be understood by patients, including those with limited health literacy (European Commission, 2009a). The European Union requires in-depth user tests on the package leaflets’ comprehensibility before marketing authorization is granted (European Commission, 2009b; Van Dijk et al., 2014).

In addition, in many countries the pharmacist composes a prescription label with directions for medication use, comprising of information related to the individual patient (name/address/date or birth), medication (name, strength, and amount of active substance), and health care professionals involved (prescriber, pharmacy) (Lester et al., 2013). In the Netherlands, the pharmacist attaches this label to the medication package for each prescription, and dispenses the product with additional verbal instructions (van Hulten et al., 2011). In other countries, such as Germany, patients do not get personalized prescription label instructions, and physicians are not obliged to write specific dosage or auxiliary instructions on the prescriptions (Botermann et al., 2016).

The prescription label serves as an independent, comprehensible information source supporting patients’ correct medication use which in turn could facilitate their medication therapy (Webb et al., 2008). Prescription labels contain dosage instructions and auxiliary instructions (Wolf et al., 2007). Dosage instructions describe how patients should use the medication, the intake frequency, and the number of units per intake (e.g., “*take two capsules twice* daily”). Auxiliary instructions consist of warnings (e.g., “*Do not drink alcoholic beverages*”) and advices (e.g., “*take with food or milk*”). Currently, the size and design of medication packages limit the amount of available space for prescription labels which means that only the most essential instructions are presented in a concise way. The combination of limited space, and the complexity of information needed to inform patients about proper use of medication, challenges the pharmacist to compose a prescription label instruction that is both informative and comprehensible. In contrast to the package leaflet, there are, as far as we know, no guidelines that provide directions on how to compose comprehensible instructions for the prescription label (European Commission, 2009b).

Research has shown that prescription labels may cause problems because they are often misinterpreted, which may lead to incorrect medication use (Davis et al., 2006a; Davis et al., 2006b; Wolf et al., 2006; Bailey et al., 2009; Wolf et al., 2010; Wolf et al., 2011; Bailey et al., 2014). One of the causes for misinterpretation is the wording of the instructions, which appears to be too difficult for patients, especially for those with limited health literacy (Beckman et al., 2005; Shrank et al., 2007; Bailey et al., 2012; Koster et al., 2014). Health literacy can be defined as the skills to obtain, understand, and use health information in order to enhance health, well-being, and active involvement in medical decision making (Sorensen et al., 2012). Research shows approximately 25% of the Dutch population has limited health literacy (Consortium, 2012). Limited health literacy is associated with negative health outcomes and poor ability to understand instructions of prescribed medication (Davis et al., 2006a; Lo et al., 2006; Wolf et al., 2007). Therefore, prescription labels instructions should be presented in an unambiguous and clear way (Bailey et al., 2009). Moreover, it is important to take health literacy into account when evaluating the comprehensibility of prescription label instructions [c.f., (Patel et al., 2018)].

However, it is unknown how specific textual elements in prescription label instructions (e.g., the presentation of numbers or use of plain language) and patients’ health literacy skills are related to their comprehension. Although Samaranayake and colleagues (Samaranayake et al., 2018) conducted a narrative review in which they discuss studies focusing on patient-related (age and literacy) and medication-label related factors (i.e., the use of icons and graphics, and the format of the label) that affect the comprehensibility of prescription labels instructions, an overview of specific textual elements associated with better comprehension of prescription label instructions was not provided. Knowledge of which textual elements facilitate patients’ comprehension of prescription label instructions, especially those with limited health literacy, will provide concrete directions for health professionals on how to optimize their medication prescriptions.

Moreover, research of Borgsteede and Heringa (2019) showed a substantial proportion of Dutch prescription label instructions was too complex, and could be optimized by rephrasing them. For example, of the 100 most frequently used dosage instructions, 23.8% were instructions for multiple times a day use without specifying the time of day (e.g., “1 tablet 2 times a day”), and 13% contained general instructions or instructions to “take as needed.” Arguably, as the instructions were about one-third of the total, the potential improvement on a population level is significant. To improve the comprehensibility of prescription label instructions, it is important to study how the textual elements as well as patients’ health literacy are related to correct interpretation of prescription label instructions. In this study, we aim to analyze, optimize, and implement comprehensible prescription label instructions in the Netherlands by using a mixed-methods approach, and to provide a study protocol for other countries to develop comprehensible prescription label instructions.

## Material and Methods

### Design

**Figure 1:**
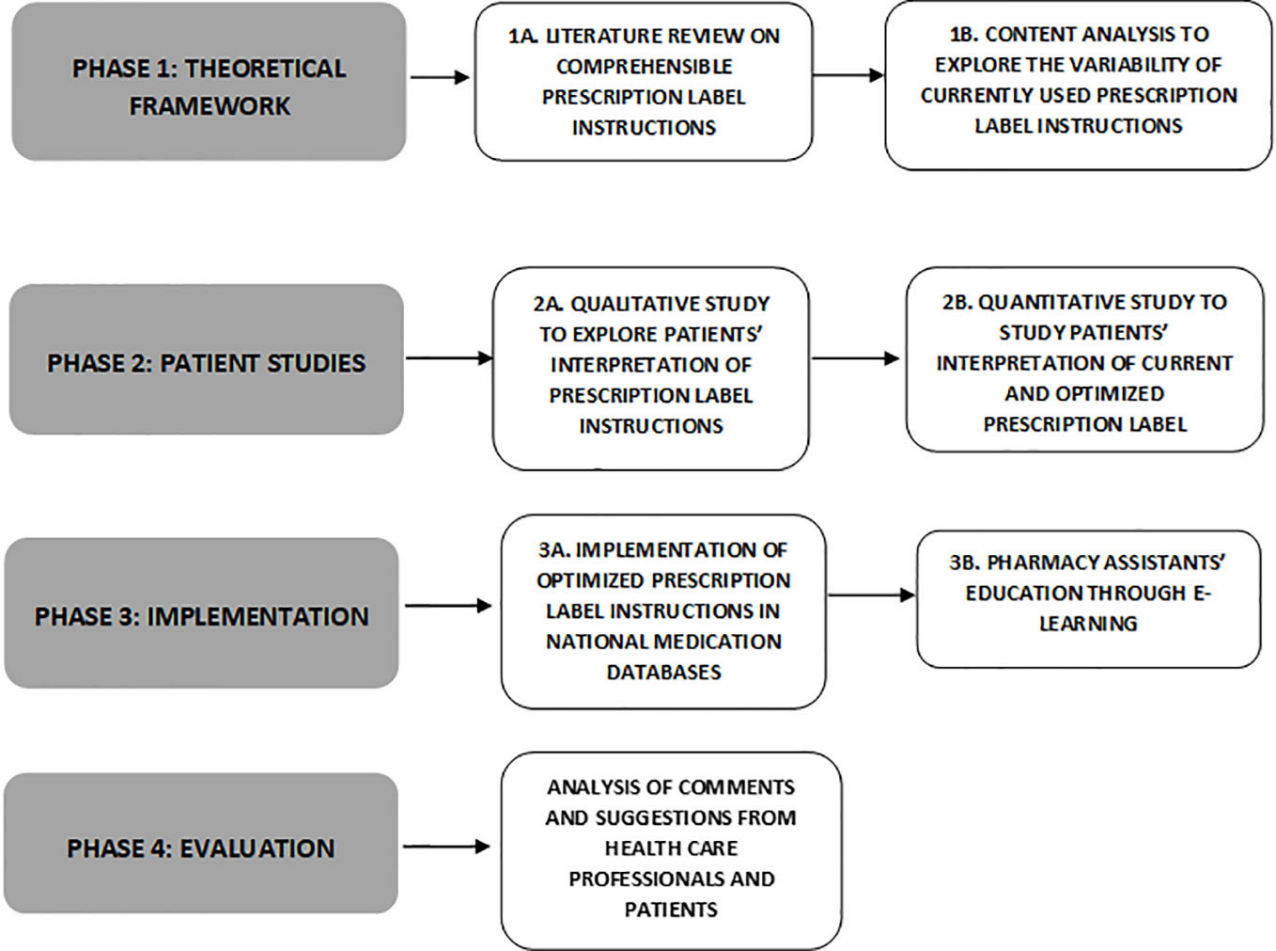
Flow chart of the four phases research on the study, development, implementation, and evaluation of comprehensible prescription label instructions.

The study consists of four phases (see **Figure 1**) and started in January 2017. Data collection for the literature review and content analysis (phase 1) and patient studies (phase 2) have been finished, and the implementation of outcomes is ongoing. In December 2021 we expect to finish Phase 4.

We have tested the comprehension of prescription label instructions in eight pharmacies across the Netherlands and included patients with different education levels and health literacy levels. Based on the studies in the four phases, we optimized prescription label instructions, and implemented and evaluated them in primary care in the Netherlands. Prescription label instructions have been optimized and implemented in the national medication databases which are used to prescribe and dispense medication. Also, a guideline has been provided on how to compose comprehensible prescription label instructions.

By using a mixed-methods approach and with the supervision of a multidisciplinary expert panel, we investigated this problem from three different perspectives, i.e., literature, linguistics, and patients. It is recommended to involve the target group when designing and evaluating health information (van Beusekom et al., 2018). Therefore, we included patients within the patient studies, with respect to health literacy. The mixed-methods approach enabled us to investigate the comprehensibility of prescription label instructions using both qualitative and quantitative research methods, in which we included patients’ interpretations to expand and strengthen the study conclusions (Schoonenboom and Johnson, 2017).

### Setting

Dutch community pharmacies use pharmacy information systems with clinical decision support. These systems contain a centrally maintained table with codes and corresponding dosage instructions. Moreover, they contain a table with predefined auxiliary labels for all available medicines (Borgsteede et al., 2019). In general, a prescriber provides patients with dosage instructions, which are a part of an electronic prescription. Next, a pharmacist processes the prescription and can either use a predefined dosage code, or use free text for the dosage instructions on the prescription label. Auxiliary labels are automatically printed on prescription labels, although the pharmacist has the discretion to overrule the labels in individual cases. Both the prescriber and pharmacist explain to patients how to use the medication. Written information is also provided by means of the package leaflet of the manufacturer and a patient-centered information leaflet.

### Health Literacy

We have included a representative sample of patients including both patients with limited and adequate health literacy skills. The interviewer followed a training on communication with patients with limited health literacy to recruit them for this study, and to support them in finishing the interview. We recruited pharmacies for the interviews which serve a diverse patient population with respect to health literacy. The Pharos Dutch Centre of Expertise on Health Disparities supported in selecting these pharmacies. To assess health literacy levels among patients, two assessment tools were used: The Newest Vital Sign in Dutch [NVS-d; (Fransen et al., 2014)], and The Recognizing and Addressing Limited Pharmaceutical Literacy (RALPH; (Koster et al., 2018; Vervloet et al., 2018).

### Expert Panel

In all phases, an expert panel has been consulted. The panel consisted of primary health care professionals (pharmacists, physicians), health literacy experts, an expert in linguistics, experts in pharmacy practice research, a patients’ representative, and pharmacists working with the national medication databases. The expert panel met prior to each phase. Results of each phase have been presented to and commented on by the expert panel. After implementing our results, the expert panel will maintain their efforts concerning comprehensible prescription label instructions by annual evaluation of reports. The underlying guidelines will be revised in consultation with the expert panel every 5 years.

### Phase 1a (Theoretical Framework): Literature Review on Comprehensible Prescription Label Instructions

Rationale: International studies have investigated the comprehensibility of prescription label instructions. The results show different textual elements impact the comprehensibility of prescription label instructions. Also, patients’ interpretations of specific textual elements might differ, due to their health literacy. The systematic literature review was conducted in 2018 and provided directions on how prescription label instructions can be optimized according to current scientific insights.

Aim: To identify the role of textual elements and health literacy on the comprehensibility of prescription label instructions.

Data collection: Electronic searches of published literature were conducted in four databases: PubMed, Embase, PsychINFO, and SmartCat. These databases were searched without time restrictions in English and Dutch language *via* the strategies outlined in **Table 1**.

**Table 1:** Search strategy in electronic databases.

Database	Search components	Search
**PubMed**	Comprehension AND drug labeling OR prescription label	((comprehension[MeSH Terms]) AND drug labeling[MeSH Terms]) OR prescription label[Title]
**PubMed**	Comprehension OR misunderstanding AND prescription label OR medication label	“Comprehension” (Mesh) OR misunderstanding (tiab)) AND (prescription label* (tiab) OR instruction label* (tiab) OR medication label* (tiab) Field: Title
**SmartCat**	Comprehension OR misunderstanding AND medication label OR prescription label	su: comprehension OR su:misunderstanding) AND (kw:medication label* OR kw:prescription label*
**PsychINFO**	Comprehension OR misunderstanding AND prescription label OR medication label OR instruction label	comprehension OR misunderstanding) AND (prescription label* OR medication label* OR instruction label*
**Embase**	Comprehension OR misunderstanding AND prescription AND label OR medication AND label OR instruction AND label	(‘comprehension’/exp OR misunderstanding:ti,ab) AND (prescription:ti,ab AND label*:ti,ab OR (medication:ti,ab AND label*:ti,ab) OR (instruction:ti,ab AND label*:ti,ab))

Studies were included if they discussed the comprehensibility of prescription label instructions and addressed textual elements of the prescription label instructions, such as medical jargon, abbreviations, the spelling of numbers (alphanumerical or numerical), and complexity and precision of dosage instructions. Studies focusing solely on the relation between the comprehensibility of prescription label instructions and the use of icons, fonts, format of the label, the way of printing, or other graphical elements, were excluded. Due to technical reasons of the printing systems, we could only adapt textual elements in prescription label instructions in this project. Moreover, one reviewer checked reference lists of the retrieved articles, in order to include relevant articles.

Analysis: We identified textual elements that impact the comprehensibility of prescription label instructions. We reported on the study characteristics, specifically the health literacy skills of the included patients, and quality of selected publications.

Outcome: We provided an overview of textual elements which facilitate or hinder the comprehension of prescription label instructions, complemented with recommendations to improve the prescription label instructions in practice.

### Phase 1b (Content Analysis): Explore the Variability of Currently Used Prescription Label Instructions

Rationale: In phase 2, prescription label instructions had to be presented to patients, that needed to be realistic in the context of medication use, yet had a variety in textual elements and topics to study differences in patients’ interpretations. Therefore the systematic literature review was combined with a content analysis in which we analyzed the variation in the topics and wording of Dutch prescription label instructions. This content analysis has been conducted in 2018.

Aim: To explore the variation in the topics the wording of Dutch prescription label instructions by performing a content analysis. This analysis informed the composition of prescription label instructions used in phase 2 (patient studies).

Data collection: We collected the currently used prescription label instructions from two national medication databases (i.e., PharmaBase from Health Base Foundation and G-Standard from The Royal Dutch Pharmacists Association).

Analysis: All prescription label instructions were analyzed on the presence of textual elements (based on the findings of the systematic literature review), and their topics (dosage instruction, auxiliary label warning, or auxiliary label advice).

Outcome: We gained insights into the frequency of textual elements used in prescription label instructions as well as the frequency in prescription label instructions’ topics.

### Phase 2a (Patient Studies): Patients’ Interpretations of Prescription Label Instructions

Rationale: To make prescription label instructions comprehensible for all patients, it is important to examine how patients interpret prescription label instructions and which textual elements facilitate or hinder patients’ interpretation. Therefore, currently used prescription label instructions and potential improvements were presented to patients with limited and adequate health literacy. Their interpretations were explored thematically. This study has been conducted in 2017.

Aim: To explore patients’ interpretations of currently used prescription label instructions and potential improvements for the prescription label instructions, and their opinion on potential improvements.

Data collection: We included patients, aged 18 years and older, who could read, and speak Dutch (although fluency was not required). Patients were approached in the waiting area of pharmacies serving a diverse patient population with respect to health literacy. Exclusion criteria were: (1) visual impairments, not resolvable by reading glasses, and (2) lack of understanding of the Dutch language. A total of 39 patients were included for in-person semi-structured interviews. We developed an interview guideline. Patients’ health literacy skills were measured subjectively and objectively, using two instruments respectively: The Recognizing and Addressing Limited PHarmaceutical Literacy (RALPH) interview guide, and The Newest Vital Sign in Dutch [NVS-d; (Fransen et al., 2014)]. The Netherlands Institute for Health Services Research (Nivel) and the Utrecht Pharmacy Practice network for Education and Research (UPPER) developed the RALPH instrument (Koster et al., 2018; Vervloet et al., 2018).

We included a varied patient group according to age, gender, mother tongue, education level, and health literacy level by purposive sampling. The researcher followed a course at the Pharos Dutch Centre of Expertise on Health Disparities on effective communication with patients with limited health literacy in order to approach and interview patients with limited health literacy skills.

Patients received verbal information about the study, and an information sheet with a short description of the study. In consultation with the Medical Ethics Committee, we concluded that written informed consent was preferred, but verbal—audiotaped—consent was possible to diminish the participation barrier for patients with limited health literacy. Interviews were held in pharmacies’ consulting rooms and were audio-recorded. At the beginning of the interview, sociodemographic characteristics were registered. Subsequently, patients interpreted seven medication labels in a random order, one by one. Medication labels were designed using a number of prescription label instructions consisting of various textual elements and varying in complexity (based on the insights of phase 1a and 1b). After choosing prescription label instructions, we combined those instructions into seven realistic prescription label instructions together with existing medication names. Prescription labels instructions were attached to medication packages of the specific pharmacy, together with the name of the medication in order to enhance the labels’ credibility. To simulate a realistic prescription label, we chose medication that is frequently prescribed in Dutch primary care, such as non-steroidal anti-inflammatory drugs, amoxicillin proton-pump inhibitors, beta-blockers, and statins (Stichting Farmaceutische Kerngetallen (SFK), 2017). Patients had to explain the prescription label instructions in their own words. Patients interpreted dosage instructions and auxiliary instructions by responding on the question: “What does this [prescription label instruction] mean?” To test their interpretation of the dosage instructions, patients were asked to demonstrate the moment and amount of medication consumption on a time schedule with candy resembling medicines. This way, interpretation and demonstration were measured independently. All patients received the currently used version of the prescription label and an optimized one. Patients were asked which label they preferred.

Analysis: Audio records of the interviews were transcribed verbatim and analyzed by the researcher based on a coding scheme (see **Table 2**). Patients’ answers were analyzed in terms of correctness. The various interpretations of patients were also explored thematically.

**Table 2:** Coding scheme for the patient study in phase 2a.

			Correctness of the given answers: yes/no
*Respondent ID*			
*Health literacy (RALPH)*			
*Health literacy (NVS-d)*			
Which version of the medication labels were presented to the patient?	1.		
	2.		
	3.		
	4.		
	5.		
	6.		
For each presented medication label: How did the respondent interpret the prescription label instruction? *Verbatim answers of respondents are cited here*	1.		
	2.		
	3.		
	4.		
	5.		
	6.		
Demonstration of the dosing instructions (only for dosing instructions on the medication schedule) *The interviewer will present a visual medication schedule to the respondent in which the respondent will demonstrate his interpretation of the dosage instructions.* *>> Insert photo of the medication scheme.*			

Outcome: Based on the initial analysis of data, the systematic literature review, and the content analysis, prescription label instructions were optimized.

### Phase 2b (Patient Studies): Patients’ Comprehension of Current Versus Optimized Prescription Label Instructions Through an Experimental Study

Rationale: In order to test whether prescription label instructions are comprehensible for patients, it is important to test patients’ comprehension of the currently used and the optimized versions among patients with different levels of health literacy. This study has been conducted in 2017.

Aim: To study patients’ interpretations of currently used and optimized prescription label instructions.

Data collection: Six prescription label instructions with a variation in textual elements and topics were chosen. Realistic prescription label instructions were composed conform phase 2a [i.e. choosing medication that is frequently prescribed in Dutch primary care (Stichting Farmaceutische Kerngetallen (SFK), 2017)]. Both the currently used and the optimized prescription label instructions were presented randomly to a sample of patients (3 currently used and 3 optimized prescription label instructions). Based on power calculation (power = 80%, α = 5%), 158 patients were recruited (79 respondents per test group). Patients were approached in the waiting area of pharmacies serving a diverse patient population with respect to health literacy, mother tongue, and education levels. Patients aged 18 years or older, who could read and speak Dutch were included. Exclusion criteria were: (1) visual impairments, not resolvable by reading glasses, and (2) lack of understanding of the Dutch language.

Patients received verbal information about the study, and information sheet with a short description of the study. In consultation with the Medical Ethics Committee, we concluded that written informed consent was preferred, but verbal—audiotaped—consent was possible to diminish the participation barrier for patients with limited health literacy. Sociodemographic characteristics were registered at the beginning of the interview. We aimed to have a representative patient group according to age, gender, mother tongue, education level, and health literacy level.

All interviews were audio-recorded. Primary outcome was the interpretation of the use of the medication according to the instructions on the medication label. Patients interpreted dosage instructions and auxiliary instructions by responding on the question: *“What does this [prescription label instruction] mean?”* To test their interpretation of the dosage instructions, patients were asked to demonstrate the moment and amount of medicine consumption on a time schedule with candy resembling medicines. This way, interpretation and demonstration were measured independently.

Considering the population, we categorized patients’ interpretations using response categories: the researcher chose the best fitting response category in order to cluster the answers within categories for further analysis. Thus, we did to not steer patients’ interpretations into the predefined response categories and prevented we would miss unexpected patients’ interpretations. Similar to phase 2a, we used the RALPH instrument (Koster et al., 2018; Vervloet et al., 2018) and NVS-d (Fransen et al., 2014) to measure patients’ health literacy skills.

Analysis: Correct interpretation of the prescription label instructions were determined by reviewing patients’ verbatim responses. Patients’ answers were analyzed based on a coding scheme (see **Table 3**) and their interpretations were also be explored thematically. In addition, the impact of patients’ health literacy on the correct interpretation of prescription label instructions were determined.

**Table 3:** Coding scheme for the patient study in phase 2b.

		Correctness of the given answers: yes/no
*Respondent ID*		
*Health literacy (RALPH)*		
*Health literacy (NVS-d)*		
Which version of the medication labels were presented to the patient?	1.	
	2.	
	3.	
	4.	
	5.	
	6.	
For each presented medication label: How did the respondent interpret the prescription label instruction? *Verbatim answers of respondents are cited here*	1.	
	2.	
	3.	
	4.	
	5.	
	6.	
Demonstration of the dosing instructions (only for dosing instructions on the medication schedule) *The interviewer will present a visual medication schedule to the respondent in which the respondent will demonstrate his interpretation of the dosage instructions.* *>> Insert photo of the medication scheme.*		

Outcome: Based on this quantitative study, the proportion of correct interpretation of textual elements in current and optimized prescription label instructions were determined, and were related to patients’ health literacy skills.

### Phase 3a and 3b: Implementation

Rationale: We have developed a guideline, a handbook, and a freely-accessible e-learning course on composing comprehensible prescription label instructions. These materials have been developed in 2018.

Aim: To implement knowledge about comprehensible prescription label instructions in pharmacy practice.

Method: Implementation has been done by developing a guideline and handbook on composing comprehensible prescription label instructions, and developing a freely-available e-learning, and an automated implementation via a national medication database.

Guideline and handbook to compose comprehensible prescription label instructions: Based on the findings in phases 1 and 2, we formulated recommendations in a guideline on composing comprehensible prescription label instructions. We also provided a handbook for pharmacy technicians which helps them to compose comprehensible prescription label instructions.

Pharmacy assistants’ education: Pharmacy assistants have been educated in composing comprehensible prescription label instructions through a freely-accessible e-learning course. This course was based on the recommendations of the guideline. Learning goals of this e-learning were:

To create awareness of misinterpreting prescription label instructions, especially for patients with limited health literacy skills.
To teach pharmacy technicians to translate dosage instructions of prescriptions into comprehensible prescription label instructions.

Pharmacy Information System: In the Netherlands, prescription label instructions used in Pharmacy Information Systems in primary care are from one of two national drug databases: Pharmabase or G-Standard. These databases are owned by Health Base Foundation (Pharmabase), and G-Standard (Royal Dutch Pharmacists Association), with pharmacies using Pharmabase data covering about 55% of the pharmacies in the Netherlands (Bahar et al., 2017). For auxiliary label instructions, optimized prescription label instructions based on the guideline were implemented in the Pharmabase in January 2018, and for the G-Standard in March 2020. Optimized dosing instructions cannot be fully implemented in the software of Pharmacy Information Systems due to technical requirements, and is supported by the guideline and e-learning course.

### Phase 4: Evaluation

After implementing the optimized prescription label instructions, comments and suggestions from health care professionals and patients will be analyzed. The Health Base Foundation manages the Pharmabase and receives comments and suggestions by phone and email from pharmacists, pharmacy assistants, general practitioners, and patients. The comments will be ordered according to type of prescription label instructions (dosage instructions/auxiliary label), topic, and textual elements. Comments will be analyzed systematically to identify similar underlying factors associated with limited comprehension of prescription labels, and suggestions for improvement will be reviewed and discussed with the expert panel. If necessary, additional qualitative and quantitative studies will be performed in pharmacy practice to compose comprehensible prescription label instructions. We will continue to optimize the prescription label instructions by considering reports from the pharmacy practice. Also, the guideline will be updated continuously, as new insights arise. The evaluation of comments will be performed in January 2021.

### Ethics and Dissemination

The patient studies have been approved by the Ethical Committee of the Amsterdam UMC, location VU Medical Centre, The Netherlands. Interview transcripts were pseudonymized before analysis. All collected data were preceded by participants’ verbal—audiotaped—informed consent, and preferably written informed consent too. Results of this research are expected to be disseminated through peer-reviewed publications, conference presentations, a PhD thesis, and presentations for interested stakeholders.

## Discussion

We proposed a mixed methods study to analyze, optimize, and implement comprehensible prescription label instructions in the Netherlands. Our study consisted of four phases, in which a systematic literature review and a content analysis on prescription label instructions textual elements and topics were conducted in the first phase (1a and 1b). To compose comprehensible prescription label instructions, patients’ perspective should be included. Moreover, it is important to take health literacy into account when evaluating the comprehensibility of prescription label instructions (Wallace et al., 2008). Therefore, in the second phase qualitative and quantitative patient studies were performed (2a and 2b). We used both subjective and objective instruments to measure their health literacy skills reliably. In the third phase, the results were implemented in a national medication database. In the fourth phase, results will be evaluated and further improved by using experiences from patients and pharmacists.

With this study, we optimized prescription label instructions, and implemented knowledge in pharmacy practice using a mixed-methods approach. We believe our approach is pertinent, as we examined the comprehensibility of prescription label instructions from different perspectives: literature, linguistics, and patients. In each phase, we consulted an expert panel, consisting of experts in the fields of health care, health literacy, linguistics, pharmacy practice research, the patients’ perspective, and the national medication databases. Studying and optimizing prescription label instructions with a multidisciplinary team adds value to this study, and supported the implementation. Therefore, we believe that our approach is unique, and shows how health professionals can provide the most essential instructions about medicine use in a comprehensible way. In addition, the results can also be applied to the verbal instructions prescribers and pharmacists provide to patients.

The European Commission published a revision of the Guideline on the readability of the label and package leaflet of medicinal products for human use in 2009 (European Commission, 2009a). This has been an important step in acknowledging the patient’s perspective in designing and evaluating medication information, and has contributed to improved readability and user testing. However, these EU guidelines do not apply to prescription label instructions. These labels are designed for individual patients, that use individual dosage regimens for their medication. We do believe that comprehensibility of prescription label instructions should be tested too by user-testing, in a similar procedure as the Guideline of the European Commission (Van Dijk et al., 2014).

Moreover, research of Borgsteede and Heringa (2019) showed a substantial proportion of Dutch prescription label instructions was too complex, and could be optimized by rephrasing them. In this study, we collected data about the comprehensibility of dosage instructions, auxiliary label warnings or advices, and how they are interpreted by patients with limited and adequate health literacy using a mixed methods approach. This allowed us to gain insight into the type of textual elements that were too complex for patients, and specifically for those with limited health literacy. Additionally, the qualitative data provided insights in how patients’ interpretation differs from the interpretation of health care professionals. In addition, by testing of currently used and optimized prescription label instructions, we gained insight in which textual elements could be improved. A guideline for testing prescription label instructions should be composed, and, we believe that this study protocol initiates a first step.

This study will also reveal which prescription label instructions are too difficult to understand because the instructions are too complex to be put on a prescription label. The number of words for prescription label instructions is generally limited, and it is not possible to refer to contextual information (e.g., about the disease). As a result, this study might also give suggestions for verbal instructions about the medication use given by the prescriber and pharmacist or written instructions in a patient-centered information leaflet. Integration and adaptation of all information sources into comprehensible patient information will support patients in therapy adherence, in particular for patients with limited (health) literacy.

### Strengths and Limitations

A limitation of the study is that no research was included on graphical elements on prescription label instructions. Due to the fact that Dutch printing systems do not facilitate printing symbols on the medication label, and our aim to implement our findings directly, we focused on the wording of prescription label instructions. Additional elements could support the comprehension of prescription label instructions. A systematic review of Sletvold et al. (2019) showed that pharmaceutical pictograms are useful for patients that are normally at risk for non-adherence to medication therapy (Sletvold et al., 2019). Also, Katz et al. concluded a combination of symbols and textual information were more effective for patients’ understanding than the use of symbols only (Katz et al., 2006). Therefore, it is important to investigate the formulation of prescription label instructions, so that combining comprehensible prescription label instructions and graphical elements could strengthen patients’ comprehension even more.

Another limitation of this this study, is that the use of printed prescription label instructions varies between countries: although this is common practice in many Western countries, this is not always the case in developing countries (U.S. Congress and O.O.T.A., 1993). We believe this protocol will identify general problems in the comprehension of information regarding medication use, and give a direction how these problems can be solved. Therefore, this research is also relevant for the countries that do not use prescription label instructions yet, as the results will enable health professionals to give comprehensible instructions in their verbal communication.

We included experiences of patients, both with adequate and limited health literacy, and analyze them using both qualitative and quantitative methods. This study identifies general problems concerning the comprehension of medication use instructions, and provide potential solutions that will improve the comprehensibility of prescription labels instructions which can also be applied to verbal communication. Also in verbal communication, clear communication is hampered by limited health literacy (Howard et al., 2013).

In conclusion, this study protocol describes a method to compose and implement comprehensible prescription label instructions. This will lead to better comprehension for patients, which in turn might lead to better adherence.

## Ethics Statement

The studies involving human participants were reviewed and approved by the Ethical Committee of the VU University Medical Center, Amsterdam, the Netherlands. The patients/participants provided their written and verbal informed consent to participate in this study.

## Author Contributions

EM, CMJH, LD, JR, and SB wrote and drafted the protocol. CMJH, LD, GB, MJ-G, JR, and SB acquired funding for this study. EM, CMJH, LD, GB, CH, MJ-G, JV, MV, HW, JR, and SB contributed to the development of the protocol, and read and approved the manuscript. All authors contributed to the article and approved the submitted version.

## Funding

This study is being funded by a ZonMw GGG-STIP grant, number 848022004. ZonMw is the Dutch national organization for health research and healthcare innovation. ZonMw’s main commissioning organizations are the Ministry of Health, Welfare and Sport and the Netherlands Organisation for Scientific Research. ZonMw did not influence the content of the guideline.

## Conflict of Interest

The authors declare that the research was conducted in the absence of any commercial or financial relationships that could be construed as a potential conflict of interest.

## Abbreviations

GP, General practitioner; NVS-d, The Newest Vital Sign in Dutch; RALPH, Recognizing and Addressing Limited PHarmaceutical Literacy.
